# Anomalously Faster Deterioration of LiNi_0.8_Co_0.15_Al_0.05_O_2_/Graphite High-Energy 18650 Cells at 1.5 C than 2.0 C

**DOI:** 10.1155/2018/2593780

**Published:** 2018-07-31

**Authors:** Dawei Cui, Jinlong Wang, Ailing Sun, Hongmei Song, Wenqing Wei

**Affiliations:** School of Mechanical-Electronic and Vehicle Engineering, Weifang University, Weifang 261061, China

## Abstract

Discharge rate is a key parameter affecting the cycle life of lithium-ion batteries (LIB). Normally, lithium-ion batteries deteriorate more severely at a higher discharge rate. In this paper, we report that the cycle performance of LiNi_0.8_Co_0.15_Al_0.05_O_2_/graphite high-energy 2.8 Ah 18650 cells is abnormally worse at a 1.5 C discharge rate than at a 2.0 C discharge rate. Combining macromethods with micromethods, the capacity/rate performance, electrochemical impedance spectroscopy (EIS), and scanning electron microscope (SEM) morphology of the electrodes are systematically investigated. We have found that the impedance of the negative electrodes after 2.0 C aged is smaller than that after 1.5 C aged, through EIS analysis, and the discharge rate performance of the negative electrodes after 2.0 C aged is better than that after 1.5 C aged through coin cell analysis. In addition, some special microcracks in the negative electrodes of aged cells are observed through SEM analysis, which can accelerate the side reaction between active and electrolyte and form the thicker SEI which will hinder the Li^+^ insertion and cause resistance increase. In short, the LiNi_0.8_Co_0.15_Al_0.05_O_2_/graphite-based lithium-ion batteries show better cycle life at a 2.0 C discharge rate than at a 1.5 C discharge rate which indicates that the negative electrodes contribute more than the positive electrodes.

## 1. Introduction

Lithium-ion batteries (LIB) used in electric vehicles (EV) need high energy density, high power capacity, long cycle life, and good security to achieve superior driving performance and better economic viability [1–4]. In recent years, the LiNi_(1-x-y)_Co_x_M_y_O_2_ system (denoted as NCM, where M represents metal elements such as Mn and Al) has been widely reported as cathode materials due to its many advantages, such as high specific capacity, long lifespan, and low cost [5–9]. Among this family, LiNi_(1-x-y)_Co_x_Al_y_O_2_ (NCA) is attracting much attention and becoming a promising candidate material for the positive electrode because of its highest capacity among the whole cathodes which have been used in mass [10–13].

There are many published studies on NCA-based LIB, focusing on the electrochemical characteristics [14–16], the storage performance [17, 18], the factors that affect the performance [19–21], and especially the capacity fading mechanism [22–25]. As reported, the main factor which deteriorated the cycle life at 100% depth of discharge (DOD) for the NCA/graphite system was the microcracks in the positive electrode [23–25] and these micro-cracks were mainly induced by the shrink in volume under charge and discharge operations [12]. It has great difference with the LiMn_2_O_4_/graphite and NCM/graphite systems in which the main factors causing the capacity fading were considered to be the dissolution of Mn, the increase of the polarization, and the decrease of active Li^+^ [26–28].

Electric vehicles (EV) and plug-in hybrid electric vehicles (PHEV) usually require LIB to work at higher charge/discharge currents, and therefore, it is very important for LIB to understand the fading mechanism at high charge/discharge rates. Generally, the cycle life for LIB is deteriorated at higher charge/discharge rates: the higher the rates, the worse the cycle life [29]. Whether it has the same conclusion for the NCA/graphite system is the question. From the reported papers, we have learned that the pulse discharge current had the worse effect on cycle life than the constant current for 3 Ah 18650 cells with NCA [30]. However, few studies have examined the effect of constant current discharge rates on cycle life of NCA-based 18650 cells. In the present work, the basic fading mechanisms for NCA-based 18650 cells cycled at two different discharge rates (1.5 C and 2.0 C) were studied and their cycle performance including capacity and morphology was discussed through electrode analysis. The initial reasons which affect the cycle life for the NCA/graphite-based LIB were further explained.

## 2. Experimental

The NCA-based 18650 cells used are from an automotive battery manufacturer Boston-Power Incorporation (BPI), and the details of the cells are listed in Table 1. The nominal capacity of the cells was 2.75 Ah (charge/discharge at 0.2 C). The positive electrode of the cells is NCA, the negative electrode is artificial graphite with polyethylene-based ceramic separate, and the electrolyte is a mixture of ethylene carbonate (EC), ethyl methyl carbonate (EMC), and dimethyl carbonate (DMC) with lithium hexafluorophosphate (LiPF6).

The cycle tests were conducted at two different discharge rates, that is, 1.5 C (4125 mA) and 2.0 C (5500 mA), by using a battery tester LAND (5 V/5 A). The temperature was controlled to 25°C by a high-low temperature chamber (Hongzhan, 80 L). Note that the same charging protocols, that is, constant current (CC) (0.5 C) and constant voltage (CV) with an upper voltage limit of 4.2 V and a cutting-off current of 138 mA, were employed in all discharge cases. The cycle tests were continued until the capacity retention decreases to 80% of the initial capacity.

In order to gain insights into the aging origins, we disassembled the fresh cell and the cells aged at different rates after discharging these cells to 2.5 V in an Ar atmosphere glove box, in which the moisture content was less than 0.1 ppm. The positive and negative electrodes were rinsed by DMC. After that, we assembled these harvested electrodes into new coin cells with Li metal as the counter electrode in the Ar atmosphere glove box. In terms of these reassembled coin cells, the capacity and rate performance were examined using battery tester LAND (5 V/10 mA). The impedance changes were measured in Princeton PARSTAT 4000 with an ac amplitude of 5 mV over the frequency range from 50 kHz to 0.05 Hz.

The pore size distribution and the porosity of fresh and aged cells were measured using the mercury intrusion porosimetry (MIP) with PoreMaster 33 type from Quantachrome. The MIP could measure pore diameters from a nanometer to micrometer scale, which matched well with the pore range in the electrode film sample compared to nano-computed tomography (CT) just monitoring the pore diameters above 500 nm [31, 32]. Scanning electron microscopy (SEM) was carried out to observe the surface microscopic morphology changes of the positive and negative electrodes for fresh and aged cells. SEM images were taken with ULTRA 55 type from Carl Zeiss SMT Corporation. For porosity and SEM analysis, the electrodes were washed with DMC and evaporated at room temperature.

## 3. Results and Discussion

### 3.1. Cycle Performance of Cells and Characteristics of Electrodes

**Figure 1:**
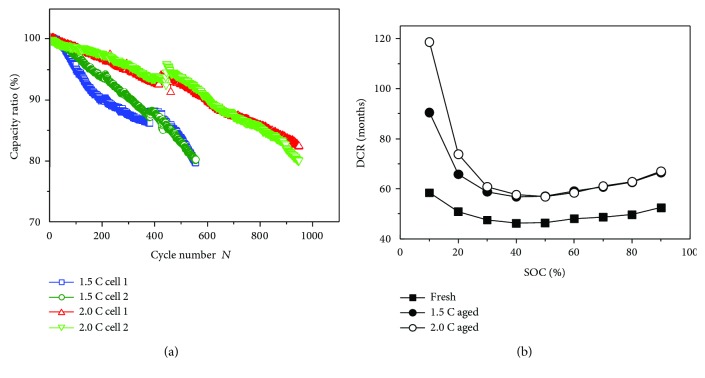
Cycle performance of NCA-based 18650 cells cycled at different discharge rates. (a) Cycle life curves; (b) direct current resistance (DCR) curves.

The cycle performance of NCA-based 18650 cells at different discharge rates is shown in Figure 1.  Figure 1(a) displays the cycle life of the cells at 2.0 C discharge rate and 1.5 C rate at 25°C, and two cells are tested for either discharge rates. It can be noted that the capacity retention at 2.0 C is better than that at 1.5 C and the former's cycle life is about twice that of the latter.  Figure 1(b) demonstrates that the direct current resistance (DCR) has increased by 20% after the cycles of one cell at various discharge rates. When the capacity decreases to 80% of the initial state, the whole output capacity/energy for 2.0 C aged cells is clearly higher than that for 1.5 C aged cells, as listed in Table 2.

**Figure 2:**
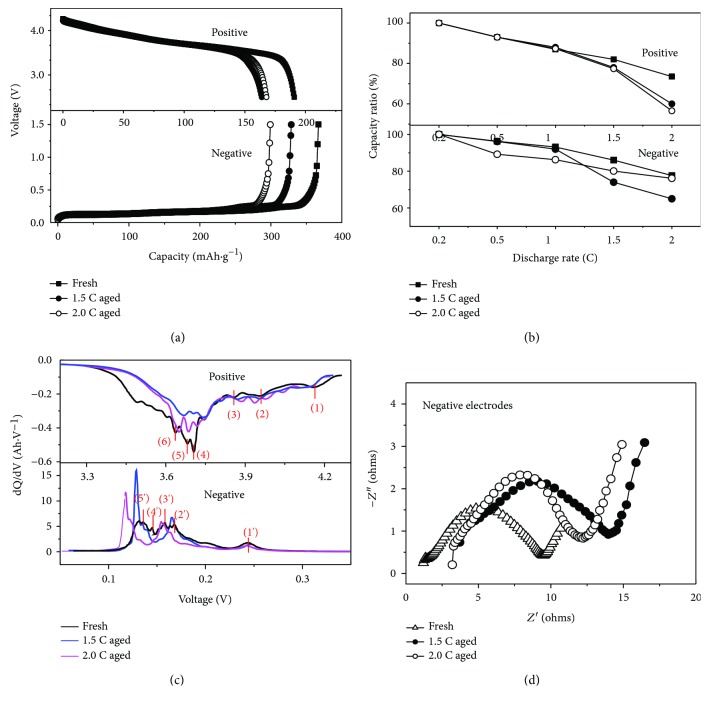
Characteristics of different electrodes for fresh and aged cells. (a) Discharge curves of different electrodes at 0.2 C; (b) discharge rate performances of different electrodes; (c) ICA curves of different electrodes; (d) and Nyquist plots of the negative electrodes.

The positive and negative electrodes for fresh and aged cells are reassembled into 2032 coin cells with Li metal as counter electrode. The capacity calibration are operated with 0.2 C CC-CV charge mode and 0.2 C CC discharge; the voltage range is 2.5 V~4.2 V for the positive electrodes and 0.005 V~1.5 V for the negative electrodes. The characteristics of different electrodes for fresh and aged cells are shown in Figure 2.

Figure 2(a) presents the discharge curves for the positive and negative electrodes. As can be seen, the capacity of the positive electrodes can remain 86% for 1.5 C aged cells and 87% for 2.0 C aged cells and this difference with cell capacity retention of 80% is derived from the different charge and discharge rates. The capacity ratio of the negative to the positive (N/P) ratio can be calculated as follows:

(1) $$\begin{array}{c} \frac{N}{P}ratio=\frac{C_{n}\times S_{n}\times r_{active-graphite}}{C_{p}\times S_{p}\times r_{active-NCA}}, \end{array}$$

where *C*_n_ and *C*_p_ are the capacity of the negative and positive electrodes, respectively; *S*_n_ and *S*_p_ are the area coating density of the negative and positive electrodes, respectively; *r*_active−graphite_ and *r*_active−NCA_ are the ratio of active material in the electrodes.

According to (1), it can be known that the N/P ratio value for 1.5 C aged cells increases to 1.19, while for 2.0 C aged cells, it decreases to 1.06, and the details are listed in Table 3. These results indicate that the negative electrodes have enough capacity balance even for aged cells so the capacity fading is attributed to the deterioration of the NCA. The above conclusion is essentially consistent with previous reports [23–25]. However, it cannot explain why the 2.0 C discharge rate is better than the 1.5 C discharge rate.

In order to explore the reason for the above phenomena, the intercalation ability of Li^+^ for the positive electrodes and deintercalation ability of Li^+^ for the negative electrodes had been evaluated based on the discharge rate performance of different electrodes for fresh and aged cells as shown in Figure 2(b). It can be figured out that the intercalation ability of Li^+^ for the positive electrodes is very similar after 1.5 C or 2.0 C aged and their capacities deteriorate by about 5% at the 1.5 C rate, about 18% at the 2.0 C rate compared with those of fresh cells. However, it can be also seen that the negative electrodes after 2.0 C aged exhibit better rate performance at 1.5 C or 2.0 C rate than those for 1.5 C aged, which implies that there is a great difference in the deintercalation ability of Li^+^ for the negative electrodes after different aging. Consequently, it can be inferred that the capacity fading of NCA/graphite-based LIB is mainly associated with the negative electrodes, which reminds us not only to consider the positive electrode's effect on capacity loss but also to attach more importance to negative electrode's effect on cycle life.

Figure 2(c) displays the incremental capacity analysis (ICA) curves of the positive and negative electrodes for fresh and aged cells at a 0.2 C discharge rate, in which each peak involves a phase reaction. For the positive electrodes, there exist six peaks in fresh and aged cells, marked separately as peak (1) to peak (6). The voltage of each peak has increased but the dQ/dV decreased for aged cells, reflecting that the phase structure do not change during the cycle. This conclusion is also similar to that of other researches analyzed through XRD and Raman methods [25], that is, the NCA crystal structure did not suffer serious damage during the cycle tests. For the negative electrodes, it can be found that there are five phase transition processes, corresponding to peak (1′) to peak (5′). The voltage of each peak has decreased and the dQ/dV increased for aged cells, especially for peak (5′), while the overall peak heights for 2.0 C aged are lower than those for 1.5 C aged, revealing that the fading of the cells for 1.5 C aged at the lower voltage is obviously induced by the negative electrodes.

**Figure 3:**
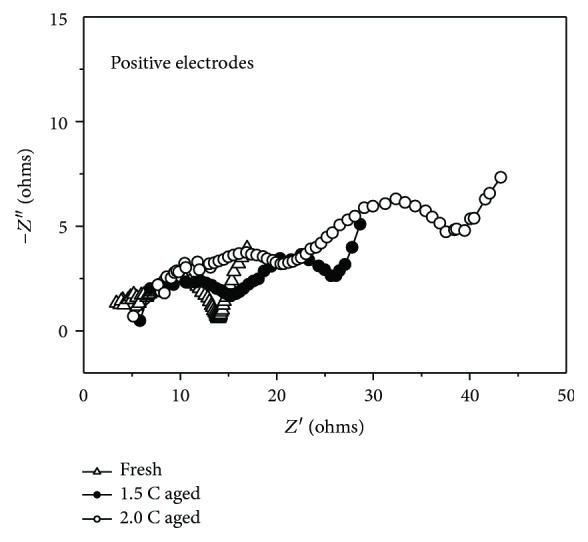
Nyquist plots of the positive electrodes for fresh and aged cells.

The impedance was measured at 50% SOC at 25°C, and the frequency range is 0.05 Hz to 50 kHz. The impedance of the negative electrodes for coin cells is shown in Figure 2(d) and that of the positive electrodes is presented in Figure 3. As seen from Figure 2(d), the impedance of the negative electrodes after 1.5 C aged is larger than that after 2.0 C aged and thus can lead to the faster deterioration of the rate performance, which is in good agreement with experimental results for rate performance in Figure 2(b). Through critical equivalent circuit model (ECM) for LIB from a relevant literature [33], the impedance was composed of ohmic resistance (*R*_o_), solid electrolyte interface resistance (*R*_SEI_), charge transfer resistance (*R*_ct_), and diffusion resistance (*R*_diff_). Accordingly, we can conclude that for aged cells, *R*_o_ increases while it is similar for 2.0 C aged and 1.5 C aged and *R*_SEI_ and *R*_ct_ increase greatly compared to fresh cells but the impedance for 2.0 C aged is smaller than that for 1.5 C aged.

### 3.2. Changes in Morphology and Porosity of Disassembled Electrodes

**Figure 4:**
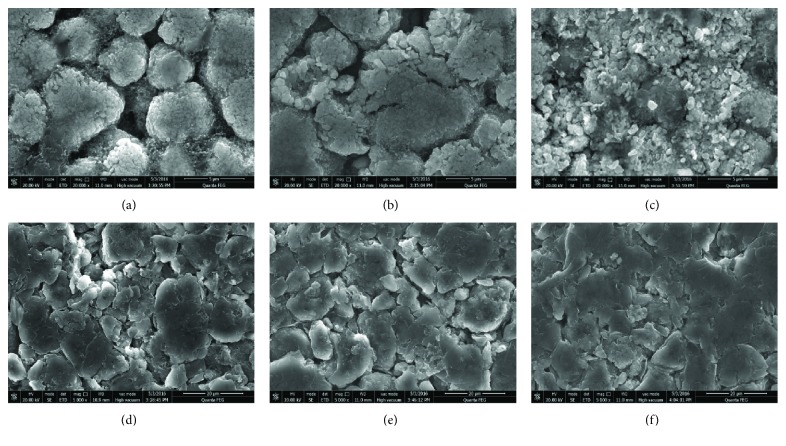
SEM images of the positive and negative electrodes for fresh and aged cells. (a) Fresh positive electrode, (b) 1.5 C aged positive electrode, (c) 2.0 C aged positive electrode, (d) fresh negative electrode, (e) 1.5 C aged negative electrode, and (f) 2.0 C aged negative electrode.

Figure 4 presents the SEM images of the positive and negative electrodes for fresh and aged cells, and the magnification for the positive electrodes is 20,000 times and 5000 times for the negative electrodes. It can be observed that there are some special microcracks in the electrodes of aged cells, not only in NCA-positive electrodes but also in graphite-negative electrodes. For the positive electrodes, these microcracks will lead to the capacity loss and resistance increase in the cells, and for negative electrodes, they will bring about the SEI reproduction which can cause the consumption of Li^+^ and resistance to increase [23–25].

**Figure 5:**
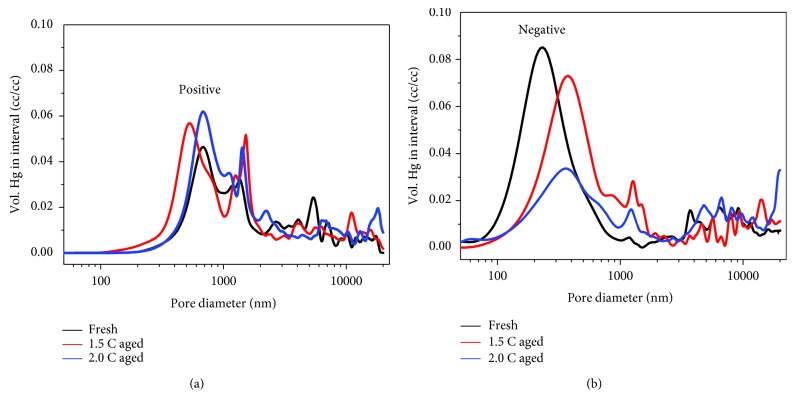
Pore distribution of the electrodes for fresh and aged cells. (a) Positive electrodes; (b) negative electrodes.

The pore distribution of the electrodes for fresh and aged cells is shown in Figure 5, and the average pore size and porosity of the electrodes are listed in Table 4. The porosity remains basically unchanged for the positive electrodes but decreases remarkably for the negative electrodes. Moreover, the average pore diameters of the negative electrodes are significantly reduced as aged. Combined with Figure 4 and relevant reference [32], it can be deduced that the microcracks will accelerate the side reaction between active and electrolyte, which is favourable to form the thicker SEI and other chemicals, and therefore hinder the Li^+^ insertion and reduce the pore diameters. However, this phenomenon is less obvious in the positive electrodes.

It is worth noticing that we have neglected the effect of electrolyte resistance on the capacity fading in the electrode analysis part. Firstly, the electrolyte has been consumed fully when we disassemble the cells and cannot get any droplet for analysis. Secondly, we have adopted the new electrolyte when we assemble the coin cells, which hinder the electrolyte effect resulting in not only the changes of composition but also the changes of ion conductive ability.

As we know, the resistance of the cells is composed of three parts and can be expressed as follows:

(2) $$\begin{array}{c} R_{cell}=R_{positive/electrolyte}+R_{negative/electrolyte}+R_{electrolyte}, \end{array}$$

where *R*_positive/electrolyte_ is the resistance of the positive electrodes and its interface resistance with electrolyte, *R*_negative/electrolyte_ is the resistance of the negative electrodes and its interface resistance with electrolyte, and *R*_electrolyte_ is only the resistance of the electrolyte.

## 4. Conclusion

To clarify the fading mechanism about the effect of different discharge rates on NCA-based 18650 cells, two different discharge rates (1.5 C and 2.0 C) were employed and the electrochemical characteristics and morphology changes of the electrodes were investigated during aged process. The capacity losses for aged cells at both discharge rates are monitored by the positive electrodes. When the capacity of the cells deteriorates to 80% of initial capacity, the residual capacity of NCA is similar, about 86% of the initial capacity at the 0.2 C rate. The negative electrodes have enough capacity balance to the positive electrodes from N/P analysis, even at the end of life which was defined as 80% of initial capacity. The difference of capacity fading for aged cells cycled at 1.5 C or 2.0 C is monitored by the negative electrodes through EIS and Li^+^ insertion and deinsertion analysis, showing that the impedance of the negative electrodes after 2.0 C aged is smaller than that after 1.5 C aged. The porosity of the negative electrodes is decreased as aged, which has blocked the Li^+^ insertion and led to the Li metal deposition even though the negative electrodes have enough balance at the end of life. The above analysis gives the initial reasons why the NCA/graphite-based LIB has better cycle life at a 2.0 C discharge rate than at a 1.5 C discharge rate, and deeper reasons should be explored in future research.

## Figures and Tables

**Table 1:** Details for NCA-based 18650 cells from BPI.

Item	Specification
Nominal capacity (Ah)	2.75
Nominal voltage (V)	3.65
Mass/g	47.0
Energy density (Wh·kg^−1^)	210

**Table 2:** Whole output capacity/energy for aged cells.

Rate	Total charge capacity (Ah)	Total discharge capacity (Ah)	Total charge energy (Wh)	Total discharge energy (Wh)
0.5 C/1.5 C	1267	1266	4888	4254
0.5 C/2.0 C	2133	2129	8271	6993

**Table 3:** Capacity and N/P ratio of the electrodes for fresh and aged cells.

Information of the electrodes		Capacity at 0.2 C (mAh·g^−1^)	N/P ratio
Fresh	Positive	190	1.09
	Negative	365.5	
1.5 C aged	Positive	164	1.19
	Negative	344	
2.0 C aged	Positive	167	1.06
	Negative	306	

**Table 4:** Average pore size and porosity of the electrodes for fresh and aged cells.

Electrodes	Type	Pore size (nm)	Porosity (%)
Positive	Fresh	312	18.0
	1.5 C aged	399	18.1
	2.0 C aged	313	17.8
Negative	Fresh	911	20.0
	1.5 C aged	574	18.0
	2.0 C aged	589	12.6

## Data Availability

The data used to support the findings of this study are available from the corresponding author upon request.
